# Nomenclature of the Teeth

**Published:** 1850-10

**Authors:** 


*Nomenclature of the Teeth.*—Mr. Tomes, in a paper "On the Structure of the Dental Tissues of Rodentia," communicated to the Royal Medical and Chirurgical Society, by William Bowman, Esq., F. R. S., May 30, 1850, and published in the London Medical Gazette, proposes the use of the following terms, as indicative of the number and arrangement of the component tissues of the teeth. He terms a tooth which has one central pulp-cavity, with tubes radiating from thence to every part of the dentine, a *dentinal system.* We see no particular objection to the term, but the inferred tubularity of dentine, though supported by high authority, we are inclined to think incorrect. When such a tooth is partially or wholly covered with enamel, he terms it a *denticle.* "A tooth composed of several dentinal systems, each of which more or less invested with enamel, and the whole united into a mass by cementum, he describes as a tooth composed of *denticles;* but if the dentinal systems are united without the intervention of enamel or cement, as a tooth composed of *dentinal systems.* If the systems are united "by a narrow strip of dentine throughout their length, the tubes belonging as much to one as to the other adjoining system," he speaks of it as a tooth made up of *confluent dentinal systems,* and if each is partially invested with enamel, as a tooth composed of *confluent denticles.*

As the paper of Mr. Tomes, to which we have referred, contains some interesting information with regard to the teeth of rodentia, we may sometime hereafter transfer it to the pages of the Journal.

